# Correction to “Macro Habitat‐Based T2‐Weighted MRI Radiomics and Deep Learning Fusion for Predicting Treatment Response and Prognosis After Neoadjuvant Chemoradiotherapy in Locally Advanced Rectal Cancer”

**DOI:** 10.1002/cam4.72056

**Published:** 2026-06-15

X. Jin , J. Xu , Y. Hu , et al., “Macro Habitat‐Based T2‐Weighted MRI Radiomics and Deep Learning Fusion for Predicting Treatment Response and Prognosis After Neoadjuvant Chemoradiotherapy in Locally Advanced Rectal Cancer,” Cancer Medicine 15, no. 5 (2026): e71773, 10.1002/cam4.71773.42071310 PMC13136072

The seventh author's name has been corrected from Sebastian Dieter to Sebastian M. Dieter to include the missing middle initial.

The online version of this article has been corrected accordingly.

We apologize for this error.

